# VLCKD: a real time safety study in obesity

**DOI:** 10.1186/s12967-021-03221-6

**Published:** 2022-01-08

**Authors:** Luigi Barrea, Ludovica Verde, Claudia Vetrani, Francesca Marino, Sara Aprano, Silvia Savastano, Annamaria Colao, Giovanna Muscogiuri

**Affiliations:** 1 Dipartimento di Scienze Umanistiche, Università Telematica Pegaso, 80143 Napoli, Italy; 2 Department of Clinical Medicine and Surgery, Endocrinology Unit, University Federico II, Via Sergio Pansini 5, 80131 Naples, Italy; 3 Centro Italiano per la cura e il Benessere del Paziente con Obesità (C.I.B.O), Department of Clinical Medicine and Surgery, Endocrinology Unit, Federico II University Medical School of Naples, Naples, Italy; 4 Cattedra Unesco “Educazione alla salute e allo sviluppo sostenibile”, Federico II, Naples, Italy

**Keywords:** Very low calorie ketogenic diet, Side effects, Obesity

## Abstract

**Background:**

Very Low-Calorie Ketogenic Diet (VLCKD) is currently a promising approach for the treatment of obesity. However, little is known about the side effects since most of the studies reporting them were carried out in normal weight subjects following Ketogenic Diet for other purposes than obesity. Thus, the aims of the study were: (1) to investigate the safety of VLCKD in subjects with obesity; (2) if VLCKD-related side effects could have an impact on its efficacy.

**Methods:**

In this prospective study we consecutively enrolled 106 subjects with obesity (12 males and 94 females, BMI 34.98 ± 5.43 kg/m^2^) that underwent to VLCKD. In all subjects we recorded side effects at the end of ketogenic phase and assessed anthropometric parameters at the baseline and at the end of ketogenic phase. In a subgroup of 25 subjects, we also assessed biochemical parameters.

**Results:**

No serious side effects occurred in our population and those that did occur were clinically mild and did not lead to discontinuation of the dietary protocol as they could be easily managed by healthcare professionals or often resolved spontaneously. Nine (8.5%) subjects stopped VLCKD before the end of the protocol for the following reasons: 2 (1.9%) due to palatability and 7 (6.1%) due to excessive costs. Finally, there were no differences in terms of weight loss percentage (13.5 ± 10.9% *vs* 18.2 ± 8.9%; p = 0.318) in subjects that developed side effects and subjects that did not developed side effects.

**Conclusion:**

Our study demonstrated that VLCKD is a promising, safe and effective therapeutic tool for people with obesity. Despite common misgivings, side effects are mild, transient and can be prevented and managed by adhering to the appropriate indications and contraindications for VLCKD, following well-organized and standardized protocols and performing adequate clinical and laboratory monitoring.

## Background

There is increasing evidence that obesity has reached an epidemic rate. In 2016, more than 1.9 billion adults over the age of 18 were reportedly overweight and more than 650 million adults were obese [1]. Obesity significantly increases the risk of developing chronic diseases such as arterial hypertension, dyslipidemia, type 2 diabetes mellitus (T2DM), coronary heart disease, cerebral vasculopathy, gallbladder lithiasis, arthropathy, polycystic ovary disease, sleep apnea syndrome, and some neoplasms [2, 3]. To achieve weight loss, one of the major challenges in the treatment of obesity is to reduce energy intake and increase energy expenditure [4]. Although various strategies have been developed to achieve this goal, the prevalence of this condition is increasing. The most frequently used dietary strategy is characterized by a reduction in fat intake and an increase in complex carbohydrates [5]. The fact that people with obesity rarely adhere to their diet is mainly because they prefer highly processed foods with simple sugars over complex/raw carbohydrates [5]. This is because foods with a high glycemic index can stimulate serotonin release, which in turn makes people feel good and promotes the onset of carbohydrate cravings [5]. Although new anti-obesity drugs are constantly appearing on the market, they still have some limitations, such as not insignificant cost, possible side effects and contraindications, which make them not suitable for all people with obesity [6]. Moreover, bariatric surgery has proven to be a useful tool for weight loss and remission of T2DM and metabolic syndrome [7]. However, there are several complications and sequelae associated with surgery, and it is limited to those individuals with severe obesity who do not have contraindications for surgery [8]. In this context, the very low-calorie ketogenic diet (VLCKD) has recently been proposed as an attractive nutritional strategy for the treatment of obesity in individuals who have already attempted to lose weight on a diet with a more balanced distribution of macronutrients without achieving the goal of weight loss. VLCKDs consist of 90% calories from fat and only 10% from carbohydrate and protein, resulting in a severely restricted diet [9]. In individuals with obesity, VLCKD has demonstrated beneficial effects on body composition, metabolic profile, and the expression of inflammation and oxidative stress genes [10–12]. The Obesity Management Task Force (OMTF) of the European Association for the Study of Obesity (EASO) carried out a meta-analysis of 15 studies to assess the efficacy of VLCKD on body weight, body composition, glycemic and lipid parameters in subjects with overweight and obesity [13]. The first finding was that VLCKD was associated with significant reductions in body weight and BMI at 1, 2, 4–6, 12, and 24 months and appeared to be associated with greater rates of weight loss compared with other diets with different energy content (i.e., low-calorie diet and very low-calorie diet) for the same duration. The second finding was that a VLCKD was associated with significant reductions in waist circumference (WC) (an expression of central adipose tissue) and fat mass, and these reductions were significantly greater than those achieved with other weight loss interventions of the same duration. The third outcome concerned blood glucose levels and Glycosilated Haemoglobin A1C (HbA1c) levels. Here, a significant reduction was found after VLCKD, without superiority compared to other weight loss measures. On the other hand, VLCKD was associated with a reduction in the homeostasis model of assessment-IR (HOMA-IR) index and an improvement in insulin sensitivity, and this effect was superior to that of other weight loss programs. The fourth finding was that a VLCKD was associated with a reduction in total cholesterol and had a greater effect in lowering total cholesterol compared with other weight loss programs. In the same vein, VLCKD resulted in a significant reduction in low density lipoproteins (LDL) cholesterol levels from baseline to post-VLCKD follow-up but did not show a superior effect compared to other weight loss diets in terms of LDL reduction. On the other hand, no change in high density lipoproteins (HDL) cholesterol was observed from baseline to follow-up after VLCKD. Interestingly, no differences were also found when we compared the mean change in HDL cholesterol between a VLCKD and other weight loss interventions. Finally, a significant decrease in triglycerides (TG) lv from baseline was associated with a VLCKD and proved to be superior to other diets [13].

Ketogenic Diet (KD) induce a metabolic state termed “physiological ketosis” by Hans Krebs, which is distinct from pathological diabetic ketosis [14]. In the past, the KD has been used to treat various diseases such as pediatric pharmacoresistant epilepsy [15]. More recently, VLCKD has undoubtedly been shown to be effective in tackling obesity [16], dyslipidemia, and most of the cardiovascular risk factors associated with obesity [17, 18]. The rapid initial weight loss is due to natriuresis and diuresis resulting from the decrease in insulin levels and the increase in glucagon levels and ketone production [19, 20]. Even after the initial diuresis, weight loss remains faster than other diets because the amount of calories is very low. In addition, because the dietary pattern is unfamiliar and the diet is perceived as temporary, patients may be able to sustain the diet better than with dietary patterns that require a longer period of time to lose the same amount of weight. Furthermore, during ketosis, subjects reported less hunger and a greater sense of satiety, a useful property to improve adherence to dietary treatments [21]. There are several hypotheses about the effect of a VLCKD on the feeling of satiety and some authors have suggested that there may be a direct effect of ketone bodies, especially B-hydroxybutyrate, on appetite suppression [22, 23]. The relative maintenance of protein mass is also an advantage, at least compared with starvation [24].

Although several studies highlighted the efficacy of VLCKD in obesity, however, the major concerns are represented by the side effects. Indeed, no studies have been carried out in subjects with obesity to specifically investigate the VLCKD-related side effects. Since the ketogenic phase of VLCKD is the most effective in weight loss and it is the phase that potentially could be associated more frequently to side effects, the primary objective of our study was to investigate the VLCKD-related side effects in obesity focusing on the time of onset and on the duration in subjects with obesity in the ketogenic phase of VLCKD. The second objective of our study was to investigate the impact of side effects on efficacy of VLCKD.

## Methods

### Subjects

We prospectively recruited 106 (12 males and 94 females, BMI 34.98 ± 5.43 kg/m^2^) consecutive patients clinically referred for weight loss treatment at the Centro Italiano per la cura e il Benessere del paziente con Obesità (C.I.B.O), Endocrinology Unit, Department of Clinical Medicine and Surgery, University Federico II of Naples (Italy), from March 2021 to September 2021. The study has been approved by the Local Ethical Committee (n. 50/20) and carried out in accordance with the Code of Ethics of the World Medical Association (Declaration of Helsinki) for experiments that involved humans. The aim of the study was clearly explained to all the study participants and a written informed consent was obtained.

Inclusion criteria were: age 18 years or older, BMI ≥ 30 kg/m^2^, naive subjects, i.e. who had not already tried treatment with anti-obesity drugs or bariatric surgery. Exclusion criteria were: type 1 diabetes mellitus, latent autoimmune diabetes in adults, T2DM on insulin therapy, pregnancy and breastfeeding, kidney failure and severe chronic kidney disease, liver failure, hearth failure (NYHA III–IV), respiratory insufficiency, unstable angina, a recent stroke or myocardial infarction (< 12 months), cardiac arrhythmias, eating disorders and other severe mental illnesses, alcohol and substance abuse, active/severe infections, frail elderly patients, 48 h prior to an elective surgery or invasive procedures and a perioperative period, rare disorders such as porphyria, carnitine deficiency, carnitine palmitoyltransferase deficiency, carnitine-acylcarnitine translocase deficiency, mitochondrial fatty acid β-oxidation disorders, and pyruvate carboxylase deficiency.

### Anthropometric measurements and physical activity

Anthropometric measurements were assessed at baseline and at the end of ketogenic phase. Measurements were performed between 8 a.m. and 12 p.m. and all the subjects were measured after an overnight fast. The anthropometric measurements were performed by the same operator, according to the International Society for the Advancement of Kinanthropometry (ISAK 2006). All the anthropometric measurements were taken with subjects only wearing light clothes and without shoes. Body weight was determined to the nearest 0.1 kg while using a calibrated balance beam scale (Seca 711; Seca, Hamburg, Germany) as well as height was measured to the nearest 0.5 cm with a wall-mounted stadiometer (Seca 711; Seca, Hamburg, Germany). In each subject, weight and height were measured to calculate the body mass index (BMI) [weight (kg)/height^2^ (m^2^)]. BMI was classified according to World Health Organization’s criteria with normal weight: 18.5–24.9 kg/m^2^; overweight, 25.0–29.9 kg/m^2^; grade I obesity, 30.0–34.9 kg/m^2^; grade II obesity, 35.0–39.9 kg/m^2^. WC was measured to the nearest 0.1 cm with a no stretch tape measure at the natural indentation or halfway between the lower edge of the rib cage and the iliac crest if no natural indentation was visible, according to the National Center for Health Statistics. Finally, the Weight Loss Percentage (WLP) was calculated using the following formula: WLP (%) = [(Starting Weight−Current Weight)/Starting Weight] × 100. Measurements were taken at baseline and at each end step of the VLCKD protocol. Participants who habitually exercised at least 30 min per day (YES /NO) were defined as physically active.

### Laboratory assay

In a subgroup of 25 subjects with obesity we assessed biochemical parameters. Blood samples were collected by venipuncture between 8 a.m. and 10 a.m. after an overnight fast. Samples were then transferred to the local laboratory and handled according to the local standards of practice. Insulin, glucose, HbA1C, lipid profile, electrolytes, uric acid, liver enzymes, and renal function were measured. The HOMA-IR [fasting glucose (mmol/l) × fasting insulin (mU/ml)/22.5] was also calculated for each subject, as previously detailed [25]. The Glomerular Filtration Rate (GFR) was calculated as follows: eGFR (ml/min/ 1.73 m^2^) = 175 × serum creatinine ^−1.234^ × age ^−0.179^ (× 0.742 if female) (× 1.212 if black) [26]. Ketosis was confirmed by the detection of acetoacetate in urine using commercially available urine reagent strips (Ketur test, Roche Diagnostics, Switzerland).

### Nutritional intervention

Subjects who met the inclusion criteria underwent to the VLCKD with the use of replacement meals following a protocol consisting in three stages: active, re-education, and maintenance. The replacement meals used for all subjects were from the same company. After the anthropometric assessment, the diet was prepared by qualified nutritionists and prescribed by the endocrinologist. The VLCKD provided a total daily energy intake of < 800 kcal depending on the quantity and quality of the preparations. The breakdown of macronutrients was as follows: ≃ 13% glucides, generally less than 30 g/day; ≃ 43% protein, daily protein intake of about 1.2–1.5 g/kg ideal body weight, ≃ 44% lipids, olive oil predominating. The VLCKD was based on protein from high biological value preparations derived from peas, eggs, soy and whey. Each protein preparation consisted of approximately 18 g protein, 4 g carbohydrates, 3 g fat (mainly vegetable oils with a high oleic acid content) and provided approximately 100–150 kcal. The weight loss program was structured in several phases. During Phase 1 (21 days), patients consumed 4–6 protein preparations (depending on ideal body weight) and low-carbohydrate vegetables, establishing the state of ketosis. In subsequent phases, the state of ketosis was still maintained. During Phase 2 (30 days) 1/2 of the meals provided (lunch and/or dinner) were gradually replaced by meals based on natural proteins (meat/fish/eggs/soy). The ketogenic period (Phases 1–2), which provided ≃ 600–800 kcal/day, was about 50 days in total. As it is a very low calorie diet, it is recommended to provide patients with micronutrients (vitamins, such as complex B vitamins, vitamins C and E, minerals, including potassium, sodium, magnesium, calcium and omega-3 fatty acids) according to international recommendations.

### Side effects assessments

The assessment of side effects was carried out through a questionnaire, periodic physical examination and laboratory assessment. The questionnaire was formulated reporting all the side effects already known to be associated with KD although in other setting of subjects i.e. migraine, dry mouth, dizziness, low blood pressure, visual disturbances, low blood sugar, lethargy, halitosis, diarrhoea, constipation, vomiting/nausea, hyperuricemia, urolithiasis, gallbladder disease, hair loss [13, 27]. It has been proposed a preliminary version of the questionnaire that was first tested in 10 patients, who were asked to comment on any aspect (content, wording and choice of answer). Questions that were ambiguous, misunderstood or rarely answered were reformulated. This resulted in a final version of 15 questions. This list of 15 potential side effects was administered and it included headache, dry mouth, dizziness, low blood pressure, visual disturbances, low blood sugar, lethargy, halitosis, diarrhoea, constipation, vomiting/nausea, hyperuricemia, urolithiasis, gallbladder disease, hair loss and whether the diet was stopped early (and why) than the end of the protocol. All questions used nominal variables (YES/NO) and were completed with information on the day of onset and duration of symptoms. Finally, information was also collected on how the symptom was managed and whether drugs and/or supplements were taken. Subjects were screened for side effects at the end of ketogenic phase.

### Statistical analysis

Continuous variables are expressed as mean ± standard deviation (SD) when normally distributed. Categorical variables are expressed as numbers and percentage (%). Variations were analyzed through the paired t-test for normally distributed variables. The p values were considered significant at p < 0.05 with 95% confidence interval. Statistical analysis was performed according to standard methods using the Statistical Package for Social Sciences software 26.0 (SPSS/PC; SPSS, Chicago, IL, USA).

## Results

Between March 2021 to September 2021, a total of 106 (12 males and 94 females; BMI 34.98 ± 5.43 kg/m^2^) subjects aged 39 ± 13.82 years underwent to the VLCKD and were included in the analyses. The main clinical characteristics of the study population are reported in Table 1. WC was 106.16 ± 14.20 cm while waist to hip ratio (WHR) was 0.88 ± 0.08. Most of the participants were sedentary (78, 73.6%). The prevalence of cardiometabolic diseases were the following: 2 (1.9%) subjects with T2DM, 9 (8.5%) with hypertension, 19 (17.9%) with dyslipidaemia, 19 (17.9%) with hypercholesterolaemia and 7 (6.6%) with hypertriglyceridaemia.

**Table 1:** Demographic and clinical characteristics at baseline

Parameters	Subjects (N = 106)
Gender	
Male	12 (11.3)
Female	94 (88.7)
Age	39 ± 13.82
BMI (kg/m^2^)	34.98 ± 5.43
WC (cm)	106.16 ± 14.20
HC (cm)	120.53 ± 10.8
WHR	0.88 ± 0.08
Physical activity	
Sedentary	78 (73.6)
Moderate	28 (26.4)
CVD	
T2DM	2 (1.9)
Hypertension	9 (8.5)
Dyslipidaemia	19 (17.9)
Hypercholesterolaemia	19 (17.9)
Hypertriglyceridaemia	7 (6.6)

Data are expressed as mean ± SD or n (%)

*BMI* body mass index, *WC* waist circumference, *HC* hip circumference, *WHR* waist-to-hip-ratio, *CVD* cardiovascular disease, *T2DM* type 2 diabetes mellitus

## Safety

Table 2 shows the side effects that occurred in our population, their onset and duration, and any medical treatment that they took to relieve side effects.

**Table 2:** Side effects occurring during ketogenic phase

Parameters	N (%)	Time to onset from the beginning of VLCKD ± SD (days)	Duration (mean ± SD) (days)	Medically treated N (%) and which remedy N (%)
Headache	48 (45.3)	4.23 ± 4.99	7.43 ± 8.84	19 (17.9)9 (47.36) ibuprofen 9 (47.36) paracetamol 1 (5.26) indomethacin
Dry mouth	41 (43.5)	3.83 ± 3.22	19.95 ± 10.35	–
Dizziness	17 (16)	6.12 ± 7.26	9.35 ± 7.37	–
Hypotension	19 (17.9)	4.68 ± 4.69	11.31 ± 10.99	–
Visual disturbances	5 (4.7)	5.40 ± 4.72	8 ± 5.43	–
Low blood sugar	1 (0.9)	2	1	–
Lethargy	49 (46.2)	4.66 ± 4.40	15.9 ± 9.6	–
Halitosis	49 (46.2)	2.90 ± 1.52	22.06 ± 8.24	18 (17) 15(83.33) chewing gum 2 (11.11) oral spray 1 (5.55) mouthwash
Diarrhoea	13 (12.3)	13.31 ± 11.48	10.08 ± 11.48	–
Constipation	30 (28.3)	11.2 ± 16.20	16.37 ± 8.95	8 (7.5) 8 (100) osmotic laxative
Vomiting/nausea	16 (15.1)	4.81 ± 4.94	4.19 ± 3.25	2 (1.9) 1 (50) metoclopramide 1 (50) antacid
Hyperuricemia	11 (10.4)	–	–	8 (7.5) 8 (100) allopurinol
Urolithiasis	0	–	–	–
Gallbladder disease	0	–	–	–
Hair loss	16 (15.1)	15 ± 9.75	15 ± 20.19	3 (2.8) 3 (100) hair supplement

Data are expressed as mean ± SD or n (%)

**Fig. 1:**
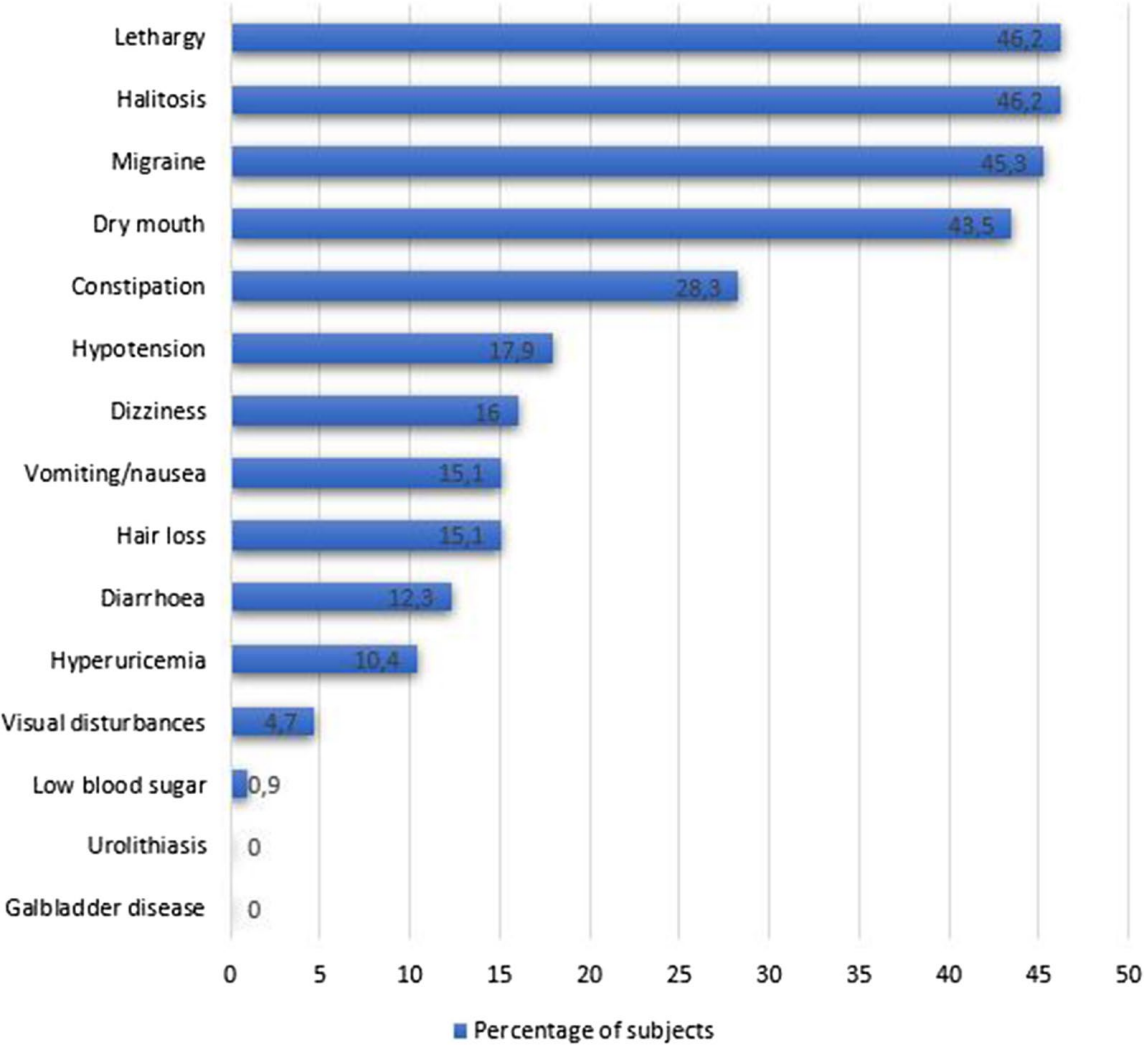
Differences in weight loss percentages from baseline to end of ketogenic phase

Regarding the kidney function, there was no significant change between GFR from baseline to the end of ketogenic phase (94.13 ± 19.00 mL/min *vs* 89.00 ± 20.83 mL/min; p = 0.123) (Table 3). With regard to liver function, we observed significant increase and decrease in AST e ALT levels, respectively (AST 20.50 ± 6.60 U/L *vs* 20.92 ± 6.32 U/L; p = 0.022, ALT 23.43 ± 9.85 U/L *vs* 22.90 ± 12.15 U/L; p = 0.001). Lastly, subjects showed a significant reduction in mean GGT from 17.82 ± 6.48 U/L to 14.72 ± 5.25 U/L (p = 0.003). No significant changes were detected in terms of serum potassium (4.41 ± 0.30 mmol/L *vs* 4.43 ± 0.33 mmol/L; p = 0.452) and serum calcium levels (9.70 ± 0.75 mg/dL *vs* 9.90 ± 0.61 mg/dL; p = 0.056) (Table 3). A significant increase of serum sodium levels has been detected (140.34 ± 2.72 mmol/L vs 140.53 ± 2.22 mmol/L; p = 0.001) Finally, there were no differences in terms of WLP (13.50 ± 10.88% *vs* 18.18 ± 8.91%; p = 0.318) in subjects that developed side effects and subjects that did not developed side effects (Fig. 1).

**Table 3:** Clinical and laboratory differences between baseline and the end of ketogenic phase

Parameters	Baseline	End of phase 1	p value
Weight (kg)	94.38 ± 17.34	87.29 ± 15.99	< 0.001
BMI (kg/m^2^)	34.98 ± 5.43	32.35 ± 5.02	< 0.001
WC (cm)	106.16 ± 14.20)	99.24 ± 13.57	< 0.001
HC (cm)	120.53 ± 10.81	115.91 ± 9.70	< 0.001
WHR	0.88 ± 0.08	0.86 ± 0.09	< 0.001
Blood Glucose (mg/dL)	88.04 ± 8.95	82.60 ± 10.08	0.072
Insulin (mg/dL)	17.35 ± 13.83	8.05 ± 5.48	0.286
HOMA—IR	3.80 ± 2.79	1.74 ± 1.29	0.332
Tot Chol (mg/dL)	170.20 ± 40.77	144.72 ± 30.61	< 0.001
LDL Chol (mg/dL)	101.95 ± 29.11	81.40 ± 29.91	0.142
HDL Chol (mg/dL)	52.24 ± 12.17	49.86 ± 13.11	0.018
TG (mg/dL)	88.95 ± 30.77	86.14 ± 20.57	0.235
GFR (mL/min)	94.13 ± 19.00	89.00 ± 20.83	0.123
Creatinine (mg/dL)	0.77 ± 0.11	0.82 ± 0.16	< 0.001
Azotemia (mg/dL)	30.44 ± 8.94	34.89 ± 10.60	0.001
Uricemia (mg/dL)	5.29 ± 1.45	6.23 ± 1.69	0.054
AST (U/L)	20.50 ± 6.60	20.92 ± 6.32	0.022
ALT (U/L)	23.43 ± 9.85	22.90 ± 12.15	0.001
GGT (U/L)	17.82 ± 6.48	14.72 ± 5.25	0.003
Calcemia (mg/dL)	9.70 ± 0.75	9.90 ± 0.61	0.056
Sodiemia (mmol/L)	140.34 ± 2.72	140.53 ± 2.22	0.001
Potassiaemia (mmol/L)	4.41 ± 0.30	4.43 ± 0.33	0.452

Data are expressed as n or mean ± SD

*BMI* body mass index, *WC* waist circumference, *HC* hip circumference, *WHR* waist-to-hip-ratio, *HOMA-IR* homeostasis model of assessment-IR, *Tot Chol* total cholesterol, *LDL-chol* low-dense-lipoprotein cholesterol, *HDL-chol* high dense lipoprotein cholesterol, *TG* triglycerides, *GFR* glomerular filtration rate, *AST* aspartate aminotransferase, *ALT* alanine transaminase, *GGT* gamma-glutamyl transferase

## Efficacy

Table 3 shows clinical and laboratory differences between baseline and the end of ketogenic phase. The weight from baseline to the end ketogenic phase was significantly reduced (94.38 ± 17.34 kg *vs* 87.29 ± 15.99 kg; p < 0.001) as well as the BMI (34.98 ± 5.43 kg/m^2^ *vs* 32.35 ± 5.02 kg/m^2^; p < 0.001). We also observed a significant reduction of waist and hip circumferences (106.16 ± 14.20 cm vs 99.24 ± 13.57 cm, p < 0.001 and 120.53 ± 10.81 cm vs 115.91 ± 9.70 cm, p < 0.001, respectively) and as can be expected there was also a reduction of WHR (0.88 ± 0.08 vs 115.91 ± 9.70; p < 0.001), from baseline to the end of ketogenic phase. Similarly, fasting plasma glucose (88.04 ± 8.95 mg/dL vs 82.60 ± 10.08 mg/dL; p = 0.072), insulin (17.35 mg/dL ± 13.83 mg/dL *vs* 8.05 ± 5.48 mg/dL; p = 0.286) and HOMA-IR (3.80 ± 2.79 vs 1.74 ± 1.29; p = 0.332) shows an improving trend despite not reaching statistically significant levels. Regarding the lipid profile, total cholesterol (170.20 ± 40.77 mg/dL vs 144.72 ± 30.61 mg/dL; p < 0.001) and HDL (52.24 ± 12.17 mg/dL vs 49.86 ± 13.11 mg/dL; p = 0.018) significantly decreased from baseline to the end of ketogenic phase. No significant changes were observed in mean LDL (88.95 ± 30.77 mg/dL vs 86.14 ± 20.57 mg/dL; p = 0.235) and mean TG levels (88.95 ± 30.77 mg/dL vs 86.14 ± 20.57 mg/dL; p = 0.235).

## Discussion

Due to the imminent increase in obesity prevalence [1], effective strategies for weight loss and weight maintenance are needed. Although bariatric surgery is an effective treatment option for patients with obesity, its invasiveness, high costs, long waiting lists and potential complications limit its widespread use [8]. Therefore, pharmacological and lifestyle-based treatments are a valuable option for most patients with obesity [6]. Although new anti-obesity drugs are constantly coming onto the market, they still have some limitations, such as not inconsiderable cost, potential side effects and contraindications, which make them unsuitable for all people with obesity [6]. In addition, dietary regimens are often characterized by limited efficacy in weight loss and poor adherence in the majority of patients [28]. Alternative dietary strategies have been introduced to achieve greater weight loss and adherence. VLCKD has been demonstrated to be a valid approach in people affected by obesity, since it promotes satiety, rapid weight loss, and muscle sparing [13]. Nevertheless, a major area of concern is the side effects of VLCKD. None of the studies carried out in subjects with obesity have been designed to specifically investigate the side effects.

In this prospective study we found the VLCKD is a safe and effective tool for weight loss and metabolic improvement in subjects with obesity. Interestingly, no severe side effects occurred in our population. In addition, those that did occur were clinically mild and they did not result in the interruption of the dietary protocol as they could be easily managed by healthcare professionals or often resolved spontaneously. The supplementation with vitamins, such as complex B vitamins, vitamin C and E, minerals, including potassium, sodium, magnesium, calcium; and omega-3 fatty acids was adequate to prevent any deficiency. Furthermore, we found that WLP was similar in those who developed side effects and those who did not (Fig. 1). Thus, the onset of side effects does not have any impact on the efficacy and on the adherence to the VLCKD.

The most common side effects that were reported were lethargy (46.2%), halitosis (46.2%), headache (45.3%), dry mouth (43.5%), constipation (28%), hypotension (17.9%), dizziness (16%), vomiting/nausea (15.1%), hair loss (15.1%), diarrhoea (12.3%), hyperuricemia (10.4%) and visual disturbances (4.7%).

Ketone bodies, which are normally produced during the active phase of VLCKD, are excreted via frequent and increased urination. This can lead to dehydration and a loss of electrolytes [29]. In a RCT comparing the efficacy and tolerability of the non-fasting KD (N = 41) and the initial fasting KD (N = 83) in children with intractable epilepsy, moderate dehydration occurred in both groups [30]. Dehydration-related disorders are mostly represented by a dry mouth, headache, dizziness/orthostatic hypotension, lethargy, and visual disturbances [22]. Thus, it is mandatory to recommend a proper water intake (at least 2 L daily), in particular during the ketogenic state. Headache was common in our patients and generally occurred in the first week. In order to relieve headache, it could be recommended to take mild analgesics as pills instead of liquid formulations because they could contain sugar that could interrupt ketogenic state. However, it should be notice that VLCKD-related headache was a short term. A considerable proportion (17.9%) of subjects also experienced hypotension thus carefully monitoring of blood pressure, increasing salt intake when there were no contraindications and the adjustment of antihypertensive drugs in subjects with hypertension is advisable during VLCKD. Another possible effect of dehydration that we have found in our population is an increase in sodiemia. This is mostly due to dehydration, although the serum sodium levels did not reach pathological values and remained in the normal ranges.

Halitosis was very frequent in our subjects (46.2%). Individuals who underwent to a VLCKD often report bad breath with a fruity smell once they reach full ketosis. Indeed, in a study of 12 healthy adults who ate four ketogenic meals over 12 h, the increase in ketone levels, and in particular the increase in acetone, acted as a predictor of ketosis [31]. Chewing sugar-free gum and/or candy and specific oral spray or mouthwash has been used as a successful strategy to manage this discomfort.

Nausea/vomiting, diarrhea, and constipation are the most common gastrointestinal (GI) side effects of a VLCKD as we also found in our study [constipation (28%), vomiting/nausea (15.1%), diarrhoea (12.3%)] and as already have been reported in studies carried out in normal weight subjects [32–34]. In an RCT, 77 healthy participants were randomized to receive a VLCKD, a low-carbohydrate diet or a low-carbohydrate diet containing 5%, 15% and 25% total energy from carbohydrates, respectively, for 3 weeks [32]. Statistically significant increase in diarrhoea and constipation severity was observed in the VLCKD group [32]. In a prospective study of 147 children with refractory epilepsy conducted to evaluate the efficacy and safety of 6 months KD treatment, the second most common side effect of dietary treatment was diarrhoea [34]. In another similar study of 12 adults with refractory epilepsy treated with KD for 4 months, mild side effects included nausea/vomiting, constipation, and diarrhoea [33]. Diarrhea could be due to defective absorption and intolerance of fat [35]. The high content of lipids can slow gastric emptying, favoring gastroesophageal reflux disease, nausea, and vomiting [35]. For the management of these symptom, it is advisable the intake of small and frequent meals, sporadic use of GI medications such as antiemetics, GI tract regulators and antacids. A decreased in water intake, fiber, and/or the volume of food can cause the onset to constipation [36]. If this was the case, it should be increased water and fiber intake and, in severe cases, the administration of low-calorie osmotic laxative is needed.

Some subjects developed hyperuricemia (10.4%) during the ketogenic phase. However, the occurrence of this adverse event is in line with what has already been reported in a systematic review of 45 studies on the safety and tolerability of the KD used for the treatment of refractory childhood epilepsy, in which hyperuricemia was reported as one of the most frequent side effects [37]. Serum uric acid is known to increase in individuals following a KD [38, 39]. To counteract this side effect, increasing water intake and, where necessary, allopurinol therapy are recommended.

Hair loss has been reported by 15.1% of enrolled subjects. Significantly negative nitrogen balance can be responsible for the hair loss that occurs during VLCKD [40]. If body protein and dietary protein mobilization are inadequate to meet the requirements, telogen effluvium is due to the low priority of hair growth of the available proteins [41]. However, hair loss is temporary, and hair regrows while weight stabilizes. Increased protein intake during VLCKD to balance nitrogen levels helps prevent or attenuate hair loss.

In addition, the relative protein excess typical of VLCKD has been of great concern among clinicians due to its potential for kidney damage. To investigate this safety outcome GFR was evaluated. GFR was not affected by dietary intervention and no differences were observed between baseline and end of ketogenic phase. Recent evidence suggest that the impact of dietary protein on renal function may depend on the protein source, with red meat intake being detrimental in a dose-dependent manner, and other protein sources such as poultry, fish, eggs and dairy products showing no such deleterious effect [42]. In addition, studies evaluating protein sources of plant origin (soy and plant derivatives) appear to show that these may even play a protective role on kidney [43, 44]. The early stages of VLCKD are based on meal replacements; the protein source of meals is whey and vegetable origin, and—when in the later stages the reintroduction of other protein sources takes place—patients are recommended to favour fish and poultry. The protein intake is never more than 1.5 g/kg/ideal body weight. It therefore seems reasonable to assume that such a dietary intervention is unlikely to have deleterious effects on kidney in individuals with obesity during the ketogenic phase.

The effect of the KD on lipid profile and cardiovascular risk is still debated due to concerns that the frequent increase in animal fat intake may counteract the beneficial effects of weight loss. Regarding the lipid profile, we found out that total cholesterol and HDL significantly decreased from baseline to the end of ketogenic phase. An important element in increasing HDL levels is physical exercise [45], and the reduction in HDL concentration we observed in our subjects is therefore probably due to the recommendation to reduce it in the ketogenic phase as it is characterized by a strong hypocaloric condition. However, a subsequent re-establishment in HDL levels can be expected in the reintroduction phase as reported in other previous studies [46, 47]. No significant changes were observed in mean LDL and mean TG levels, probably due to the prolonged ingestion of high lipid intake. In this regard, a systematic review of 107 studies found no adverse effects on serum lipid parameters, blood pressure, or fasting blood glucose in adults who followed a diet containing less than 60 g/day of carbohydrate [48], although the analysis was complicated by heterogeneity and lack of studies, particularly those that evaluated diet use for > 90 days. A 56-week study of a KD in men with obesity (N = 66) who lost 26% of their body weight found significant reductions in total cholesterol, LDL, and TG and increases in HDL [49]. The positive changes were greater in subjects with hyperlipidemia at baseline [49]. Even in studies of normal-weight subjects (N = 20) with minimal weight loss, slight to moderate increases in total cholesterol and LDL levels were seen in the KD groups [18]. These changes occurred as early as 3 weeks and appeared to return to baseline after 6 weeks in at least one study [18].

KD is also an effective tool for improving glycaemic control variables [50, 51]. In a study of 64 subjects with obesity and high blood glucose levels on a KD for 56 weeks, glucose levels showed significant improvement at the end of treatment [51]. Another study of 363 subjects with overweight or obesity investigated the beneficial effects of the low-carbohydrate ketogenic diet (LCKD) compared with the low-calorie diet in improving glycemic parameters [50]. Both treatments were associated with a reduction in blood glucose and glycated haemoglobin but changes were more significant in subjects who were on the LCKD [50]. Likewise, in our subjects, fasting plasma glucose, insulin and HOMA-IR shows an improving trend despite not reaching statistically significant levels. This is probably due to the drastic reduction in carbohydrates of ketogenic phase, which in turn reduces insulin concentrations and encourages the use of stored fat as fuel, as well as significantly reducing insulin resistance [52].

Finally, there were no differences in WLP between subjects who developed side effects and those who did not. Thus, the occurrence of side effects did not affect efficacy or compliance with VLCKD probably because they were very mild and easily managed. To our knowledge, there are no other studies in the literature that have evaluated the impact that VLCKD side effects might have on the efficacy of dietary treatment.

## Conclusions

VLCKD appears to be an ideal therapeutic tool for people with obesity, particularly those who have already tried other nutritional strategies without success and/or who have a rapid need to lose weight (people with obesity with joint diseases, people with obesity with indications for bariatric surgery, people with obesity with cardiovascular risk factors, etc.). In spite of common misgivings, side effects are mild and preventable thanks to the indications and contraindications provided for VLCKD, by following organised and standardised protocols, and by careful clinical and laboratory monitoring. For this reason, supervision by a healthcare professional is indispensable. Finally, once the goal has been achieved, it is extremely important to recommend an adequate lifestyle (physical activity and a balanced diet such as the Mediterranean diet) for maintaining weight loss in the long term.

## Data Availability

The datasets used and/or analysed during the current study are available from the corresponding author on reasonable request.

## Abbreviations

T2DM: Type 2 diabetes mellitus
VLCKD: Very low-calorie ketogenic diet
WC: Waist circumference
HbA1c: Glycosilated haemoglobin A1C
HOMA-IR: Homeostasis model of assessment-IR
LDL: Low density lipoproteins
HDL: High density lipoproteins
TG: Triglycerides
KD: Ketogenic diet
BMI: Body mass index
WLP: Weight loss percentage
WHR: Waist to hip ratio
GFR: Glomerular filtration rate
GI: Gastrointestinal
LCKD: Low-carbohydrate ketogenic diet
